# *Trans*-Translation Is an Appealing Target for the Development of New Antimicrobial Compounds

**DOI:** 10.3390/microorganisms10010003

**Published:** 2021-12-21

**Authors:** Rodrigo Campos-Silva, Gaetano D’Urso, Olivier Delalande, Emmanuel Giudice, Alexandre José Macedo, Reynald Gillet

**Affiliations:** 1 CNRS, Institut de Génétique et Développement de Rennes (IGDR) UMR6290, University of Rennes, 35000 Rennes, France; camposrdrg@gmail.com (R.C.-S.); gaetano.durso@univ-rennes1.fr (G.D.); olivier.delalande@univ-rennes1.fr (O.D.); emmanuel.giudice@univ-rennes1.fr (E.G.); 2 Programa de Pós-Graduação em Ciências Farmacêuticas, Faculdade de Farmácia and Centro de Biotecnologia, Universidade Federal do Rio Grande do Sul, Porto Alegre 90610-000, Brazil; alexandre.macedo@ufrgs.br

**Keywords:** antibiotics, ribosome, SmpB, tmRNA, *trans*-translation

## Abstract

Because of the ever-increasing multidrug resistance in microorganisms, it is crucial that we find and develop new antibiotics, especially molecules with different targets and mechanisms of action than those of the antibiotics in use today. Translation is a fundamental process that uses a large portion of the cell’s energy, and the ribosome is already the target of more than half of the antibiotics in clinical use. However, this process is highly regulated, and its quality control machinery is actively studied as a possible target for new inhibitors. In bacteria, ribosomal stalling is a frequent event that jeopardizes bacterial wellness, and the most severe form occurs when ribosomes stall at the 3′-end of mRNA molecules devoid of a stop codon. *Trans*-translation is the principal and most sophisticated quality control mechanism for solving this problem, which would otherwise result in inefficient or even toxic protein synthesis. It is based on the complex made by tmRNA and SmpB, and because *trans*-translation is absent in eukaryotes, but necessary for bacterial fitness or survival, it is an exciting and realistic target for new antibiotics. Here, we describe the current and future prospects for developing what we hope will be a novel generation of *trans*-translation inhibitors.

## 1. Introduction

Protein synthesis, or translation, is a fundamental biological process that occurs on ribonucleoprotein nanomachines named ribosomes. The bacterial ribosome is, therefore, a major antibiotic target, and many types of inhibitors can stop bacterial growth by binding its functional centers and interfering with the ribosome’s ability to synthesize proteins [1]. However, bacteria have evolved a wide set of mechanisms to resist the inhibitory effect of antibiotics, including those that target the ribosome. Indeed, resistance mechanisms have been identified for nearly every antibiotic currently in clinical use. Combined with the fact that pharmaceutical companies have not developed more than a few antibiotics recently, infections that are treatable now will probably, once again, become life threatening [2].

It is generally accepted that among the most important bacteria to target, those in the ESKAPE pathogens group (*Enterococcus faecium*, *Staphylococcus aureus*, *Klebsiella pneumoniae*, *Acinetobacter baumannii*, *Pseudomonas aeruginosa*, and *Enterobacter* spp.) are of enormous interest when it comes to drug discovery [3,4]. They are the leading cause of nosocomial infections throughout the world, and most are multidrug-resistant isolates [5]. The World Health Organization (WHO) regularly issues global reports on antimicrobial resistance (AMR) surveillance [6], and the topic has ranked in the top 10 global health issues over the past few years [4,7].

To combat this crisis, we need new antibiotics, and, most importantly, we need new classes of antibiotics with novel mechanisms of action [8]. To do this, we must first identify new molecular processes that can be targeted. Ideally, these should be conserved among pathogenic bacteria; indispensable to the survival, or at least to the fitness, of the bacteria; sufficiently specific, so that they can distinguish between bacterial species and minimize microbiotal damage; not targeted by current antibiotics; absent in eukaryotes to limit toxicity. In fact, *trans*-translation, the primary quality control mechanism for rescuing stalled ribosomes in bacteria, appears to be a perfect candidate, allowing us to target this key cellular process in a totally new way. Here, we discuss the potential of targeting this pathway with novel antimicrobial compounds.

## 2. Ribosomal Stalling: From No-Go to Non-Stop

Several phenomena can cause the production of aberrant mRNA molecules that lead to the accumulation of stalled ribosomes in bacteria. The most frequently observed are spontaneous mutations in their corresponding genes, as well as transcription defects after the RNA polymerase prematurely terminates transcription, or does not correctly transcribe the stop codon [9]. Other phenomena include mRNA degradation, caused by either endo- or 3′–5′ exo-ribonucleases, or by environmental stresses that result in chemical and physical damage [10]. “Non-stop” situations (readthrough) can also occur when a canonical stop codon is translated in the presence of non-sense suppressor tRNA [11,12], aberrant frameshifts [13], or translational error-inducing drugs [14]. In bacteria, translation initiation mainly relies on the binding of the ribosomal binding site, the Shine–Dalgarno (SD) sequence to the 3′-end of 16S ribosomal RNA. This, therefore, means that translation can start before transcription is actually complete, and that non-stop events, such as degradation, can occur both before translation starts or while the ribosome advances along the mRNA [15]. It must be noted that another type of defective translation event can also appear during certain stressful conditions (e.g., starvation), which, during translation, slow or stop ribosomes upstream from the stop codon. Due to the presence of a stop codon, this situation is called “no-go” instead of “non-stop.” Even though this process could eventually be reversed, it is problematic if it occurs for too long, as endonucleases, such as RelE (the toxin component of the type II RelE–RelB toxin–antitoxin system), will cut the mRNA within the ribosomal A site to facilitate tmRNA-mediated rescue, and conserve the energy and nutrients being used to combat stress [16]. The “no-go” then becomes “non-stop,” and triggers the same quality control mechanisms for ribosomal release. In all of these cases, the rescue of non-stop ribosomes is essential in most or all bacteria [17], suggesting that interference with non-stop quality control mechanisms is surely a promising antibiotic development path.

## 3. *Trans*-Translation Components Are Major Targets for Interference

Despite the recent discovery of several back-up systems (see [18], for a complete review), *trans*-translation is the principal and most sophisticated quality control mechanism for avoiding inefficient protein synthesis on stalled non-stop bacterial ribosomes. It mainly relies on the complex between tmRNA and SmpB, the two main actors in the process.

### 3.1. Transfer-Messenger RNA (tmRNA)

**Figure 1:**
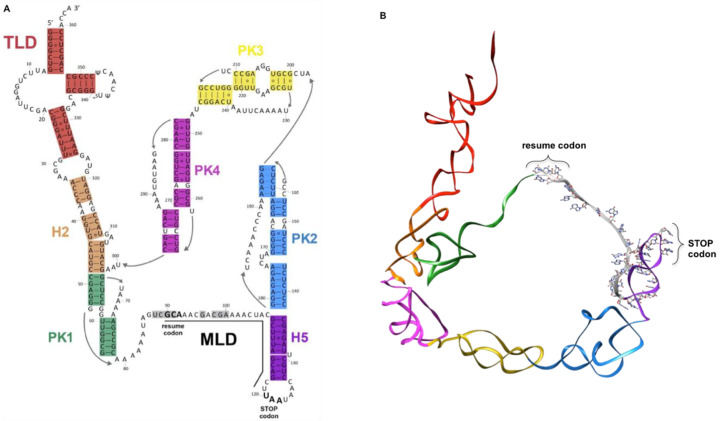
tmRNA. (**A**) Organization of the secondary structure of *Escherichia coli* tmRNA. The internal open reading frame is underlined. (**B**) 3D structure of the *E. coli* tmRNA molecule. In both panels, the tRNA-like domain (TLD, red) is followed by helix H2 (orange). The pseudoknot ring is composed of PK1 (dark green), PK2 (steel blue), PK3 (yellow), PK4 (magenta), helix H5 (purple), and the mRNA-like domain (MLD, grey). The resume and stop codons are indicated.

Having both transfer and messenger RNA functions, tmRNAs are chimeric RNA molecules that are typically 260 to 420 nucleotides in length (363 nts in *Escherichia coli*). The *ssrA* gene, which encodes tmRNA, has been found in nearly all bacterial genomes [19]. tmRNA is always first transcribed as a precursor, and it is subsequently processed at its CCA 5′- and 3′-ends [20,21,22,23]. The number of tmRNA molecules per cell has been estimated to be 500–700, roughly 5% of the total number of ribosomes, as estimated from the ratio of tmRNA-to-5S ribosomal RNA [24,25]. As with classical tRNA, the T-loop undergoes some base modifications, with the TrmA enzyme catalyzing 5-methyluridine and TruB enabling pseudouridine production [26,27]. The classic mature tmRNA is composed of a tRNA-like domain (TLD), a messenger-like domain (MLD), and a large, halo-shaped pseudoknot (PK) ring (Figure 1).

The TLD portion plays the same role as in classical tRNA; the acceptor stem is always recognized and aminoacylated by alanine tRNA synthetase (AlaRS) after recognition of a G3:U base pair, a motif also present in canonical tRNA^Ala^ [28,29,30]. The domain displays a classical T-loop, and a small D-loop without a stem. It is also devoid of an anticodon loop, since no codon will need to be recognized within the vacant decoding site of a stalled ribosome [20,26,31]. In fact, this ostensible problem is overcome by SmpB; when interacting with the TLD region, it mimics codon–anticodon recognition and allows tmRNA to accommodate into the ribosomal A site (see below). The MLD is the RNA portion that contains the internal open reading frame (ORF) of tmRNA, which encodes the aminoacidic sequence A*ANDENYALAA in *E. coli*, with the first A* being carried by the TLD. This sequence is added to the stalled protein during *trans*-translation. The tag sequence displays strong phylogenetic conservation, with the consensus sequence A*AN----ALAA. The final three alanines (AxAA) are crucial, allowing for specific recognition of the tagged protein by proteases. The nature of the RNA sequence upstream from the resume codon allows for the correct placement of the codon into the decoding center. Accordingly, mutations in this region can lead to reading frameshifts or a loss of tmRNA function [32,33,34]. In fact, the structural elements that precede the resume codon, rather than the sequence itself, are important for the reinitiation of translation [35,36,37,38,39]. Once the ORF is completely translated, the tagged peptide is specifically degraded by several proteases. In addition to this classical single-chain conformation, tmRNA also exists (in alpha-proteobacteria, cyanobacteria, and some beta-proteobacteria lineages) as a two-piece molecule, a formation caused by a circular gene permutation that splits it into two molecules [40,41]. In this case, the TLD, MLD, and PK1 are similar to those of “one-piece” tmRNA, but the loop containing the tag reading frame is broken, and there are fewer pseudoknots [42].

### 3.2. SmpB

**Figure 2:**
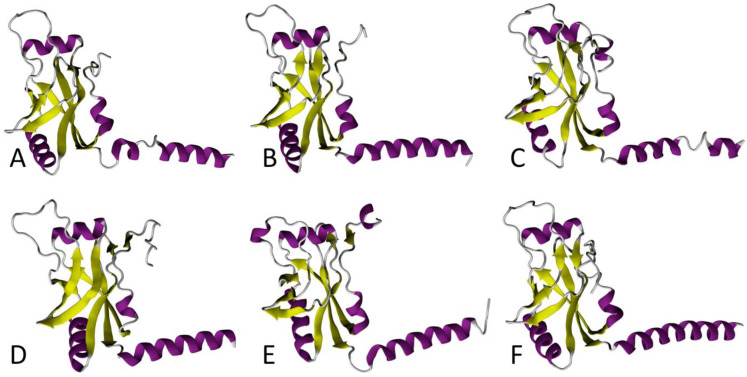
Comparison between 3D models of SmpB in ESKAPE bacteria. In each conformation, the tmRNA TLD contact region is on the left side, and the helix-shaped C-terminal end is shown to the right. The α-helices and β-strands are purple and yellow, respectively. These models of *Enterococcus faecium* (**A**), *Staphylococcus aureus* (**B**), *Klebsiella pneumoniae* (**C**), *Acinetobacter baumannii* (**D**), *Pseudomonas aeruginosa* (**E**), and *Enterobacter cloacae* (**F**) were all computed with the I-TASSER program using *E. coli* SmpB as the structural template (PDB 7ACJ).

SmpB is a small, basic protein of ~160 amino acids, encoded by the *smpB* gene. In the *E. coli* genome, it is located just upstream from the *ssrA* gene that codes for tmRNA [43]. SmpB binds to tmRNA with high affinity, and is its most important partner during ribosome rescue. In fact, in its absence, tmRNA can no longer accommodate its TLD portion into the vacant A site [44,45,46,47]. A comparison between the SmpB proteins in the various ESKAPE bacteria reveals that the six proteins conserve the same fold, but the sequence and length of the C-terminal tail differs, especially in *S. aureus*, *A. baumannii*, and *E. cloacae* (Figure 2).

The SmpB body is arranged in an oligonucleotide/oligosaccharide binding (OB) domain that folds in a classical fashion into a β-barrel made up of six antiparallel β-strands, which is also typical of other RNA-binding proteins, such as IF1 or bS1 [48,49,50]. By interacting with the tmRNA TLD, SmpB mimics the missing D-loop and anticodon stem–loop present in classical tRNA [51,52]. The interesting shape assumed by the tmRNA–SmpB complex is important for its entry into the ribosome, as it simulates the codon–anticodon pairing, which then promotes the reactivity of a cognate tRNA. Of the ~160 amino acids, the last 30 C-terminal residues form a tail, which is unstructured in solution, but folds into an α-helix during *trans*-translation. This C-terminal tail is rich in positively charged side-chain residues, essential for contacts with the tmRNA helix H5, as well as for interactions with the negatively charged nucleotides within the decoding site of the 30S ribosomal subunit [53,54,55,56,57]. Indeed, by inserting into the mRNA entry channel, the C-terminal tail is instrumental in selecting the stalled ribosomes with empty mRNA entry channels. The recent cryo-electron microscopy (cryo-EM) structures of *E. coli* tmRNA–SmpB bound to a stalled ribosome [56,57], and the previous crystallographic study of *trans*-translation in *Thermus thermophilus* [58], both show that, just as in canonical translation, the presence of the protein in the decoding center induces reorientation of nucleotides A1492 and A1493 in helix 44. Besides its main RNA-binding site on the TLD, SmpB also has a secondary RNA-binding site, which later binds the MLD to ensure that the resume codon is correctly positioned in the ribosomal A site [56]. These results confirm the long-predicted importance of SmpB in the *trans*-translation partnership [53,55,59].

## 4. The Molecular Process of *Trans*-Translation

**Figure 3:**
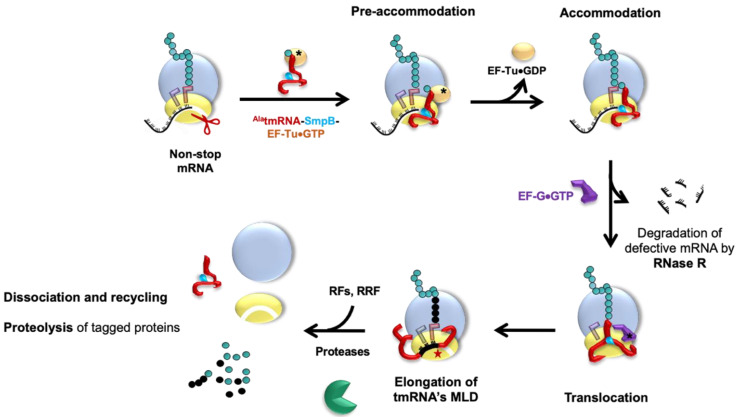
The complete *trans*-translation quality control cycle. **Pre-accommodation state:** tmRNA associates with its partner SmpB to form a complex. Elongation factor EF-Tu•GTP binds to ^Ala^tmRNA–SmpB, thereby forming the quaternary complex needed to rescue the ribosome stalled on a non-stop mRNA. To recognize these ribosomes, this quaternary complex enters the vacant ribosomal A site. There, SmpB mimics a codon–anticodon pairing while its C-terminal tail inserts into the mRNA channel. The EF-Tu•GDP is then released after GTP hydrolysis. **Accommodation**: The ^Ala^tmRNA–SmpB complex is accommodated into the A site, triggering the peptidyl transfer reaction. **Translocation:** Thanks to GTP hydrolysis, EF-G•GTP helps shift the tmRNA–SmpB into the P site. EF-G•GDP is released, and the non-stop mRNA is ejected then degraded by RNase R. **Elongation**: The tmRNA open reading frame is placed into the A site, and new tRNAs arrive at the ribosome to resume translation. **Termination**: The tmRNA–SmpB complex moves towards the E site, and the TLD and SmpB are promptly ejected. Translation of the MLD continues until translation of the tmRNA-encoded tag is terminated at the stop codon with the help of the release factors (RFs). The ribosomal subunits are then dissociated by the ribosome recycling factor (RRF), and the nascent peptide is degraded by ClpXP/ClpAP/FtsH/Tsp proteases. All of the components are now recycled and ready for a new round.

During *trans*-translation (Figure 3), the tmRNA–SmpB complex is first brought to the ribosome with EF-Tu•GTP. Stalled ribosomes are selected by SmpB, whose C-terminal tail probes the mRNA entrance channel [58]. In this pre-accommodation state, GTP hydrolysis in EF-Tu is favored, as are conformational changes in the ribosomal subunits, and this induces the accommodation of tmRNA–SmpB into the vacant ribosomal A site. After transpeptidation occurs between the stalled incomplete peptide and the tmRNA alanine, a swap between the tmRNA MLD and the non-stop mRNA allows translation to resume. The C-terminal tail of SmpB, which was involved in ribosomal vacant A site recognition, then rotates by 60° to allow the MLD to move into the mRNA channel, as well as to allow ejection of the problematic mRNA. The ribosome translates the MLD until it reaches the stop codon, after which the stalled ribosomes are recycled (see [60], for the structural details of the process). The incomplete peptides are tagged with a signal sequence that results in quick proteolysis. Two AAA+ proteolytic enzymes (ATPases associated with various cellular activities), ClpXP and ClpAP, are able to degrade the tagged proteins by converting ATP hydrolysis energy into mechanical work [61]. FtsH, a hexameric protease anchored to the internal side of the cytoplasmic membrane, is also involved in degrading a small subset of tagged proteins present in the inner membrane [43]. On the other hand, the energy-independent protease Tsp takes over the tmRNA-tagged substrates in the periplasm [62]. The problematic mRNAs are degraded by RNase R [63]. This enzyme, of 92 kDa, belongs to the RNase II superfamily, a group of exoribonucleases able to degrade the RNA molecules in the 3′ → 5′ direction [64], as well as digest various RNA substrates [65,66]. However, the details of how the ribonuclease works with the complex to promptly recognize and handle problematic mRNA is still unclear.

## 5. *Trans*-Translation as a Target for New Antimicrobial Compounds

Considering that *trans*-translation is absent in eukaryotes, tmRNA–SmpB is an especially promising target for novel antibiotics. Obviously, when it is essential to the survival of pathogenic bacteria, the *trans*-translation machinery is an excellent specific target for use in developing molecules to kill bacteria directly [35,67,68]. When non-lethal, because alternative rescue factors can take over the rescue process, deletion of tmRNA and/or SmpB induces various phenotypes, including loss of virulence or loss of antibiotic tolerance [69,70,71,72]. These hypersensitive mutants are not viable in the presence of low doses of some protein synthesis inhibitors (chloramphenicol, lincomycin, spiramycin, tylosin, erythromycin, and spectinomycin) that do not otherwise significantly affect the growth of wild-type cells [69,70,73]. Strikingly, mutants deleted for tmRNA are also more sensitive to antibiotics that do not target translation than wild-type cells, such as inhibitors of cell wall synthesis. This is probably because these drugs stress the bacteria, and this is handled more efficiently when *trans*-translation is active [74]. In all of these cases, it is possible that *trans*-translation inhibitors could be used in combination with already commercialized antibiotics, in order to diminish their minimal inhibitory concentration (MIC) in pathogens, or even to reenable the use of antibiotics no longer used because of resistance. Finally, *trans*-translation is also important for persister survival, as well as tolerance to a variety of antibiotics and stresses [75]. Despite the enormous potential and extensive research into how it works and how this pathway can be targeted for treatments against bacterial infection, there are currently no drugs on the market that use this mechanism. Since this review focuses on *trans*-translation, we will only discuss the possible strategies for specifically impairing that process via targeting tmRNA, SmpB, and/or the ribosome itself. However, we must mention that a global strategy should not overlook the possibility of altering the activity of supporting actors, such as the highly conserved aminoacyl-tRNA synthetase (AlaRS) enzyme, serine protease ClpP, or ribonuclease RNase R.

## 6. Antibiotics Targeting *Trans*-Translation: Are We There Yet?

### 6.1. Oxadiazole Compounds

**Figure 4:**
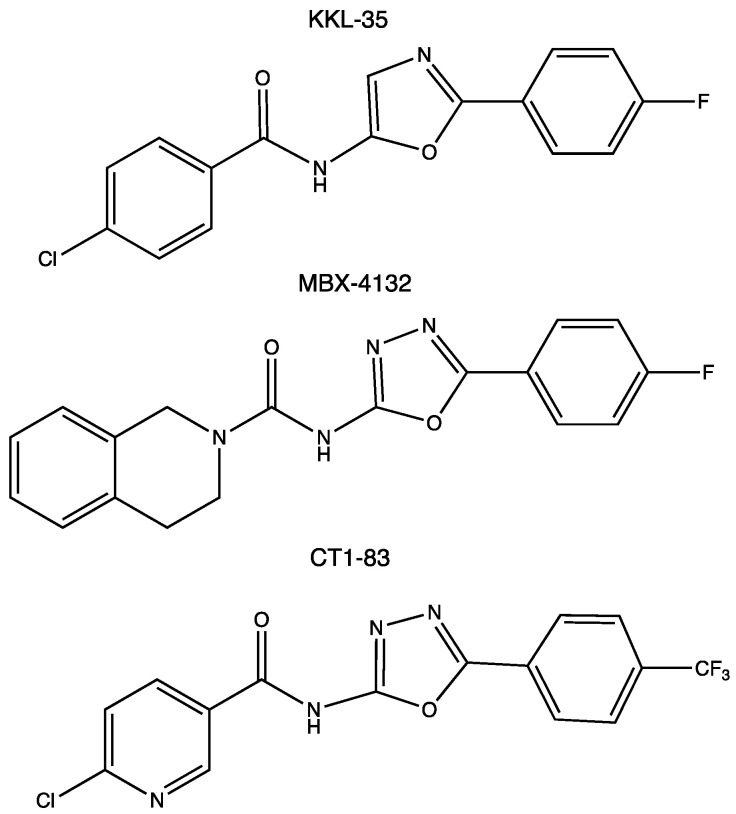
Chemical structures of the experimental oxadiazole compounds KKL-35, MBX-4132, and CT1-83.

In 2013, based on a luciferase assay, Keiler’s group performed a high-throughput screening assay on a library of 663,000 candidate compounds. This led to the identification of 1,3,4-oxadiazole and tetrazol-based compounds as broad-spectrum antibiotics that specifically inhibited the pathway [67]. The most promising compound was the oxadiazole KKL-35 (Figure 4), which displays an antibiotic effect against very distantly related bacteria, suggesting that it may have antibiotic activity against a broad spectrum of species, thus paving the way for the development of the first class of small molecules inhibiting *trans*-translation. How KKL-35 targets *trans*-translation could not be easily identified. KKL-35 binds poorly to tmRNA and SmpB, suggesting that the compound probably affects a later step in the quality control process. Indeed, later biochemical experiments, using *Mycobacterium smegmatis* and *Staphylococcus aureus* cells, highlighted KKL-2098, an analog of KKL-35 that incorporates a photoreactive azide group and a terminal alkyne moiety. KKL-2098 targets helix 89 of 23S rRNA, but in a region not targeted by conventional antibiotics. It binds to a pocket adjacent to the peptidyl transfer center (PTC), without inhibiting canonical translation [68,76]. More recently, this result was confirmed by cryo-EM (EMDB with the accession code EMD-20121). Despite a rather low occupancy, KKL-2098, cross-linked to a non-stop ribosome, binds near the PTC and significantly alters the conformation of the ribosomal protein bL27. This suggests that 1,3,4-oxadiazoles may, at least in part, inhibit *trans*-translation by preventing tmRNA–SmpB binding at the A site, or by interfering with the translocation of the complex from the A to the P site [77]. In another oxadiazole example, a *Bacillus subtilis* proteomic response library was used to show that KKL-35 and other oxadiazole derivatives induce responses that are similar to those of ionophores, which disturb metal homeostasis, and to other agents, causing oxidative stress responses. This activity could be linked to the importance of *trans*-translation in cells undergoing oxidative stress [78].

In 2017, our group developed a new double-fluorescence reporter system for the simultaneous and specific quantification of bacterial *trans*-translation, as well as proteolysis, in *E. coli* [79]. However, when we tested KKL-35, we did not observe any significant changes in fluorescence levels, despite its strong antibiotic activity, suggesting that *trans*-translation is not its only target, or that the molecule is rapidly metabolized (certainly due to amide bond fragility, see below) and the resulting products of degradation act on another target in *E. coli*. These data were supported by the fact that the inhibitory activity of KKL-35 is similar in both a Δ*arfA* strain (in which *trans*-translation is essential) and in a Δ*ssrA* strain deprived of *trans*-translation. Furthermore, in the human pathogen *Legionella pneumophila* (which causes Legionnaires’ disease), the antibiotic activity of KKL-35 is not related to the specific inhibition of *trans*-translation, as it remained active against *L. pneumophila* mutants expressing an alternate ribosome-rescue system and lacking tmRNA [80].

Because the characterization of a new antibiotic target in living cells can be slow, difficult, and treacherous (as shown with KKL-35), we recently constructed a system to detect *trans*-translation in vitro [81]. It is based on an engineered tmRNA variant that reassembles green fluorescent protein (GFP) when *trans*-translation is active. This system is, thus, adapted for the high-throughput screening of chemical compounds by fluorescence, and the limited number of reaction components allows for the direct detection of the relevant targets of *trans*-translation, which are as follows: tmRNA, SmpB, and the ribosome itself. Based on this simple system, we demonstrated that several 1,3,4-oxadiazole compounds do, indeed, inhibit *trans*-translation in vitro, though only moderately [81,82]. In KKL-35, replacing the benzene of the chloro-aryl moiety with a pyridine group (compound CT1-83, see Figure 4) results in much stronger inhibition of *trans*-translation.

However, because of the rapid hydrolysis of the amide bond of KKL-35 in liver microsomes, it cannot be used in animals. A recent structure–activity relationship (SAR) program thus led to the development of a new uriedo-oxadiazole derivate, MBX-4132 (Figure 4). This compound is much more stable and not significantly less potent, able to inhibit *trans*-translation both in vitro and in vivo, and clears multidrug-resistant *Neisseria gonorrhoeae* in infected mice [77]. While the oxadiazole strategy has been deeply studied, its cellular targets and mode of action remain uncertain, which justifies further investigation, as well as the continued search for other molecules.

### 6.2. Pyrazinamide

In 2011, it was proposed that pyrazinamide (PZA), a mainstay of anti-tuberculosis combination therapy [83], inhibits *trans*-translation [84]. Using proteomic studies, pyrazinoic acid (POA), the hydrolyzed and active form of PZA, was shown to bind to the ribosomal protein S1, encoded by the *rpsA* gene [84]. Interestingly, POA only inhibits *trans*-translation and not canonical translation, and this inhibition depends strictly on wild-type *M. tuberculosis* S1. Crystal structures of the S1–POA complex revealed that the residues Lys303, Phe307, Phe310, and Arg357 in the S1 domain directly interact with POA, and that mutations on these locations blocked the interaction with the drug, and diminished the binding between S1 and tmRNA [85]. However, the action of PZA on S1 and *trans*-translation in *M. tuberculosis* was called into question, and experiments suggest that this drug directly targets a critical player in the metabolism of coenzyme A instead [86]. A recent study seems to confirm this hypothesis, since no measurable binding between POA and S1 could be recovered, despite the use of a wide panel of biophysical methods, including nuclear magnetic resonance (NMR) spectroscopy, isothermal titration calorimetry (ITC), microscale thermophoresis (MST), and electrophoretic mobility shift assays (EMSA) [87].

### 6.3. Peptides and Oligonucleotides

Peptide aptamers (PA) are combinatorial proteins that consist of a stable scaffold protein and random amino acids designed to bind to specific targets, in order to disrupt their activity [88]. In a recent study into the ways to vaccinate and protect zebrafish against infection, PAs were developed to target SmpB in *Aeromonas veronii* [89]. These opportunistic bacteria depend on *trans*-translation for virulence, and they are commonly found in aquaculture, where they cause wound infection, diarrhea, and septicemia [90]. The aptamers directed against SmpB were selected from a PA library, and the leading aptamer PA-1 (sequence: GGVTFLVNTYPNGVQSRAGG) was shown to specifically target SmpB, and to knockdown its functioning. When PA-1 was introduced into *A. veronii*, the engineered strain was much less virulent and could be used as a potential attenuated live vaccine, thereby providing a novel strategy for preventing *A. veronii* infection [89]. A second aptamer PA-2 (sequence: IGQEWGLGVRGPLSAK) was demonstrated to interact not only with SmpB, but also with the alternative rescue factor ArfA, resulting in the dysfunction of both rescue factors [91]. Considering the expected conservation of the fold in SmpB (see Figure 2), PA-1 and PA-2 could theoretically target a wide range of different bacteria.

Another peptide strategy involves using a peptide that mimics the SmpB C-terminal tail to compete with endogenous SmpB for binding in the vacant ribosomal A site, thus preventing tmRNA recruitment and, in turn, inhibiting *trans*-translation. We showed that the peptide that corresponds to the C-terminal extremity of *E. coli* SmpB (sequence: GKKQHDKRSDIKEREWQVDKARIMKNAHR) acts as a potent *trans*-translation inhibitor in vivo [79].

Finally, the most obvious strategy is to use antisense oligonucleotides directed towards the genes encoding SmpB or tmRNA (*ssrA* gene), or towards the mature tmRNA itself. This approach is already in use in vitro by various laboratories, often as an internal control, with an antisense oligonucleotide targeting the tmRNA MLD and, thereby, very efficiently inhibiting *trans*-translation (for an example, see [92]).

## 7. Conclusions

**Figure 5:**
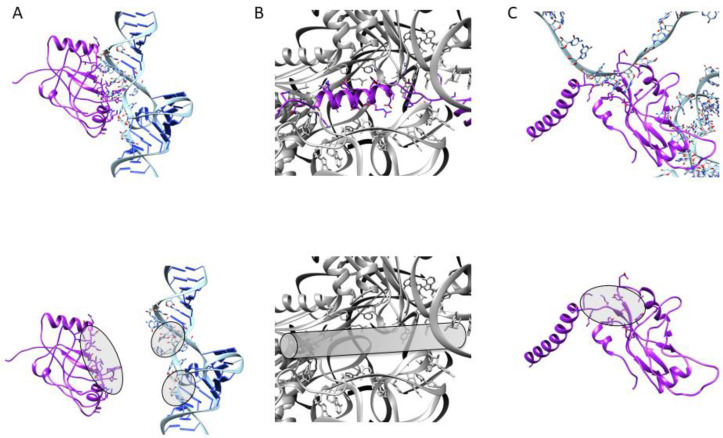
Potential anti-*trans*-translation targets. To interfere with *trans*-translation, one can (**A**) inhibit the tmRNA–SmpB interaction by targeting the binding sites of either partner; (**B**) compete with the SmpB C-terminal tail for stalled ribosome recognition; (**C**) alter the tagging process by targeting the binding site between SmpB and the MLD.

Although *trans*-translation was discovered more than 25 years ago, and has been studied carefully ever since, with several attempts made to develop molecules to target it, the only chemical family that has displayed potential activity derives from 1,3,4-oxadiazole compounds. The recent development of sensitive and selective high-throughput screening assays that target ESKAPE pathogenic bacteria will undoubtedly help us to find new scaffolds that specifically target ribosome rescue [92]. Current studies work from scratch, by screening pharmacologically active small molecules from large chemical or natural product libraries [93], or are based on rational drug design, attempting to target the interactions between tmRNA, SmpB, and the ribosome (Figure 5), as recently described in cryo-EM structural studies [56,57]. Among the interactions discussed, the most promising targets may be the TLD–SmpB interface (in order to inhibit the tmRNA–SmpB interaction before the complex enters the ribosome); the mechanism of stalled ribosome recognition (to block or compete with SmpB C-terminal tail insertion into the empty mRNA channel); the SmpB–MLD binding site, which allows resume codon registration during translocation (to impede protein tagging). The possible specific integration of such molecules within pathogenic bacteria would be an extraordinary tool in the fight against multiresistance. There is no doubt that the groundwork already laid will soon respond to this increasingly urgent antibiotic resistance emergency.

## Data Availability

Not applicable.
